# IL-5, IL-31 and systemic immune-inflammation index as biomarkers predicting severity and progression in bullous pemphigoid

**DOI:** 10.3389/fmed.2025.1657952

**Published:** 2025-10-15

**Authors:** Yanyu Zhang, Xia Liu, Lili Hou, Yan Zhao, Jiaxuan Hao, Na Zhang, Jing Tian, Xiaoqing Li, Zhiyu Liu, Hongwei Zhou, Chunyang Xu, Yuan Ren, Haizhou Zhou

**Affiliations:** ^1^Department of Laboratory Diagnosis, The First Affiliated Hospital of Harbin Medical University, Harbin, Heilongjiang, China; ^2^Department of Dermatology, The First Affiliated Hospital of Harbin Medical University, Harbin, Heilongjiang, China; ^3^Department of Clinical Laboratory, The Second Affiliated Hospital of Harbin Medical University, Harbin, Heilongjiang, China

**Keywords:** bullous pemphigoid, interleukin-5, interleukin-31, systemic immunoinflammatory index, disease severity, biomarker, ROC curve

## Abstract

**Introduction:**

Inflammatory mechanisms assume a pivotal role in bullous pemphigoid (BP). IL-31, IL-5, and the systemic immunoinflammatory index (SII) are emerging markers of inflammatory activity, but their associations with BP severity remain unclear. This investigation sought to investigate the correlation of the three aforementioned indicators with BP disease severity to determine their potential application in predicting BP severity.

**Methods:**

This study enrolled 83 BP patients and 50 healthy controls (HC). Patients were stratified into mild-moderate (MM, *n* = 23) and severe (Sev., *n* = 60) groups based on body surface area (BSA) involvement. Serum IL-5 and IL-31 levels were measured by Chemiluminescence Immunoassay (CLIA) and ELISA. The SII was calculated as platelets × neutrophils/lymphocytes. Differences between groups were assessed using non-parametric tests, correlations were analyzed using Spearman’s correlation, and predictive performance was evaluated using logistic regression and ROC curves. Five patients underwent longitudinal IL-31 and IL-5 measurement pre- and post-treatment.

**Results:**

Interleukin-31 and IL-5 levels were significantly raised in BP versus HC (median [Q1–Q3]: IL-5: 2.86 [1.22–7.10] vs. 0.79 [0.65–1.00] pg/mL; IL-31: 103.61 [77.42–146.77] vs. 86.02 [51.49–122.53] pg/mL), with further increases in Sev. versus MM groups (IL-5: 3.84 [1.57–10.63] vs. 1.35 [0.83–2.93] pg/mL; IL-31: 125.99 [91.58–173.85] vs. 65.14 [51.27–85.47] pg/mL). Post-treatment IL-31 decreased significantly in 5 patients (145.95 ± 44.25 vs. 108.62 ± 33.45 pg/mL). IL-5, IL-31, and SII positively correlated with BSA (*r* = 0.458, 0.544, 0.418). Logistic regression analysis identified elevated levels of IL-5, IL-31, and SII as risk factors for the exacerbation of BP disease. ROC analysis demonstrated the remarkable predictive capacity of IL-5, IL-31, and SII for BP disease severity.

**Conclusion:**

Interleukin-5, IL-31, and SII are promising biomarkers for assessing and predicting BP severity.

## 1 Introduction

Bullous pemphigoid (BP), a chronic autoimmune subepidermal blistering disease, manifests as cutaneous and mucosal vesicles/bullae associated with erythema. Deposited autoantibodies targeting BP180 and BP230 antigens drive bullous pemphigoid pathogenesis. The pathomechanism of the disease has been associated with complement activation, Th2-associated immune responses, and eosinophil chemotaxis (1–3).

The estimated annual incidence of BP in Asia is 2.6–7.6 cases per million people, and the disease carries a high risk of death. A timely assessment of disease severity and appropriate therapeutic measures can reduce mortality associated with BP (1, 4). Currently, the clinical assessment of disease severity in BP relies on subjective metrics such as the area of the lesion, the number of blisters, and the BPDAI score, and objective and quantifiable biomarkers are urgently needed to optimize the assessment system.

The Th2-type cytokines IL-5 and IL-31 show high levels of secretion in BP (5–7). IL-5 has been demonstrated to directly regulate eosinophil activation and lesion development, and its concentration has been shown to correlate with the number of blisters in BP (5, 8, 9). Primarily produced by Th2 cells, the cytokine IL-31 elicits itch and Th2 inflammatory responses by activating associated receptor signaling complexes (10–13). Research indicates that IL-31 contributes to inflammation in skin diseases like urticaria and atopic dermatitis and correlates with disease severity. However, its relationship to the severity of BP has not been elucidated (14–16).

The systemic immune-inflammatory index (SII), a systemic indicator of inflammation that integrates neutrophil, lymphocyte, and platelet counts, is still a gap in BP, although it has shown prognostic value in a variety of autoimmune disorders (17–19).

This study aimed to investigate the correlation of IL-5, IL-31, and SII with BP disease severity and to provide a basis for the development of an accurate assessment tool.

## 2 Materials and methods

### 2.1 Patients

The present study was conducted at the First Hospital of Harbin Medical University. Eighty-three patients diagnosed with BP and hospitalized from July 2023 to November 2024 were included in the study. The diagnosis of BP was confirmed on the basis of the patients’ clinical features; subepidermal blisters; eosinophilic and neutrophilic infiltration, as revealed by histopathological examination; and the presence of IgG autoantibodies to the skin basement membrane bands, as determined by indirect immunofluorescence assay (IIF) and ELISA for anti-BP180/BP230-specific antibodies in the blood. In accordance with the consensus among scholars regarding the diagnosis and treatment of BP, the severity of the disease was quantified using body surface area (BSA), in conjunction with lesion size and type (20). The following patients were excluded: those in a stable stage with no skin lesions; those with concomitant malignancy; pregnant and lactating women; those with immunodeficiency or other active autoimmune diseases; and those with eosinophilia, contact dermatitis, atopic dermatitis, eczema, ichthyosis, or other associated dermatologic conditions. Additionally, the study included 50 healthy controls (HC) who were matched by sex and age. All serum samples were stored at −80 °C.

### 2.2 Cytokine detection

The ELISA kit (Elabscience Biotechnology Ltd.) was used to detect IL-31, following the instructions for use. IL-5 was detected by a fully automated chemiluminescence instrument (C2000) (Hotgen Biotechnology Co., Ltd.).

### 2.3 Laboratory indicator testing

Neutrophil (NEUT), LYMPH, and PLT counts were analyzed employing the fully automated XN-9000 hematology analyzer (Sysmex, Japan).

### 2.4 Statistical analysis

Use R Studio and GraphPad Prism 10.1 for statistics and graphing. Intergroup comparisons of continuous variables employed the Student’s *t*-test for normally distributed data or the Mann-Whitney U test for non-normally distributed data. Categorical variables were compared with the Chi-square test or Fisher’s exact test, as appropriate. Associations between variables were assessed using Spearman’s rank correlation coefficient. Binary logistic regression was employed to identify determinants associated with BSA. We constructed three logistic regression models with varying levels of covariate adjustment: (1) an unadjusted model; (2) a model adjusted for demographic factors (gender and age); and (3) a fully adjusted model incorporating clinical variables and laboratory markers (cardiovascular disease, diabetes mellitus, immunomodulators, medication status, and infection). The discriminatory ability of SII, IL-31, and IL-5 for predicting BSA was assessed using receiver operating characteristic (ROC) curve analysis.

## 3 Results

### 3.1 Demographics, clinical, and serologic features

The study cohort comprised 83 BP patients (mean age 72.81 ± 9.74 years) and 50 healthy controls. Fifty-three of them suffered from cardiovascular diseases, 16 from diabetes mellitus. Prior to sample collection, 19 patients were administered glucocorticoids and immunomodulatory drugs. Healthy controls averaged 70.54 ± 11.65 years (Table 1).

**TABLE 1:** Baseline characteristics of BP patients as well as healthy controls.

Characteristics	BP n = 83	HC n = 50	*P*-value
Age, years, M ± SD	72.81 ± 9.74	70.54 ± 11.65	0.230a
**Sex, n (%)**			
Male	40 (48.19)	24 (48.00)	0.983b
Female	43 (51.8)	26 (52.00)	
**Medical history, n (%)**			
Cardiovascular disease	53 (63.86)	NA	
Diabetes	16 (19.28)	NA	
**Treatment, n (%)**			
Glucocorticosteroid glucocorticoids/ immunomodulatory drugs	19 (22.90)	NA	
Infection, n (%)	33 (39.80)	NA	

a, Student’s *t*-test; b, Chi-square test; Treatment, medication use within 1 month prior to sampling; M, mean; SD, standard deviation; BP, bullous pemphigoid patients; HC, healthy controls.

### 3.2 Patients with BP have elevated levels of IL-31 and IL-5 in their serum

**FIGURE 1:**
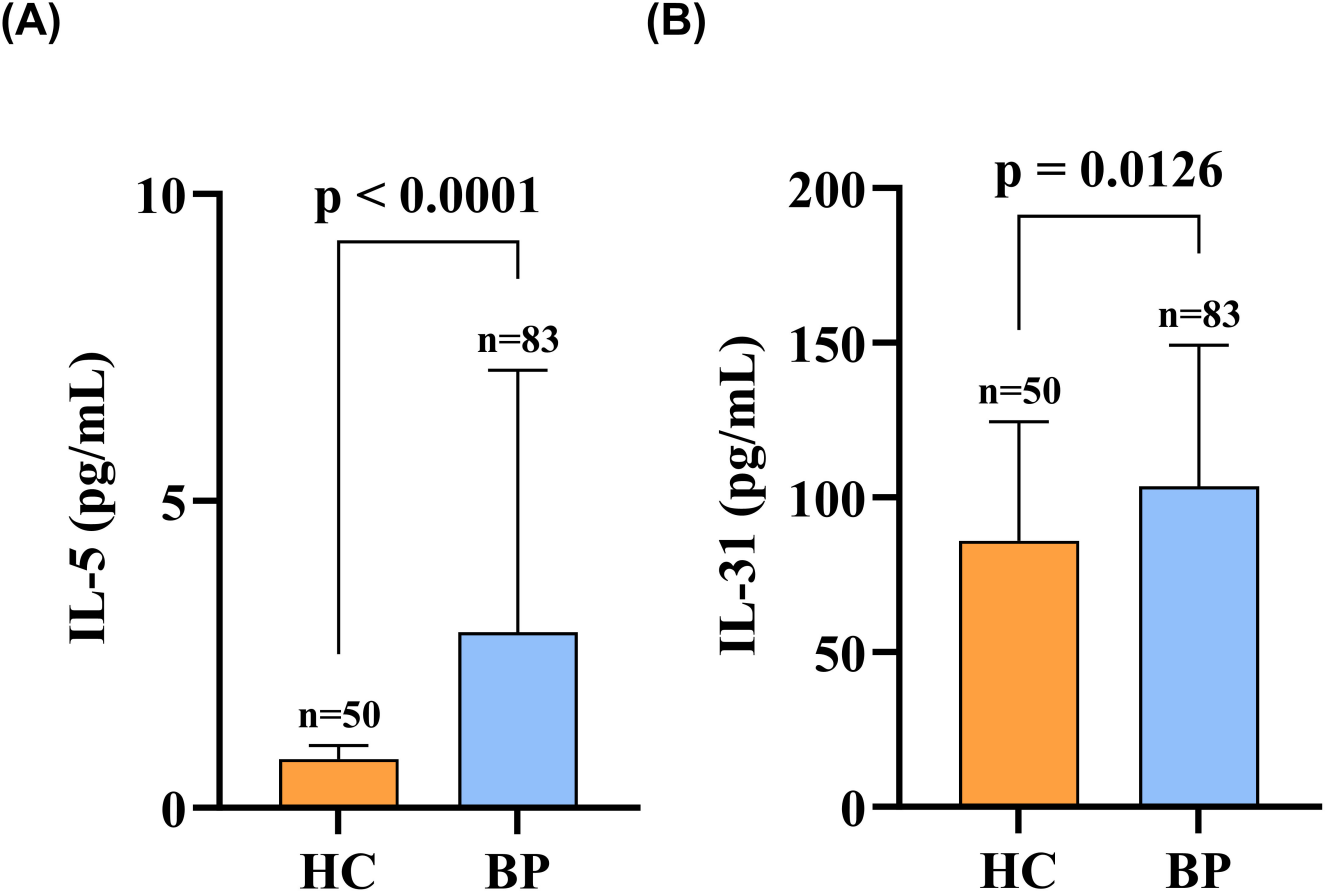
Serum IL-5 and IL-31 levels were elevated in patients with BP compared with sex- and age-matched healthy controls. **(A)** Serum IL-5 levels in patients with BP compared with healthy controls; **(B)** Serum IL-31 levels in patients with BP compared with healthy controls. Statistical analysis was performed using the Mann-Whitney test, and the differences in serum IL-31 and IL-5 levels between the two groups were statistically significant (*p* < 0.05). BP, patients with bullous pemphigoid; HC, healthy controls; IL-31, interleukin 31; IL-5, interleukin 5.

The levels of IL-31 and IL-5 were measured using ELISA and chemiluminescence, respectively, in 83 patients with BP and 50 healthy individuals. Serum IL-31 concentrations were significantly higher in BP patients [median (IQR): 103.61 pg/mL (77.42–146.77)] than in healthy controls [86.02 pg/mL (51.49–122.53)] (*p* = 0.013). Serum IL-5 concentrations were also significantly elevated in BP patients (median [IQR]: 2.86 [1.22–7.10] pg/mL) relative to healthy controls (0.79 [0.65–1.00] pg/mL; *p* < 0.001), as illustrated in Figure 1.

### 3.3 SII, IL-31, and IL-5 are associated with BSA in patients with BP

Disease severity was quantified based on BSA, and patients with BP were categorized into a mild-moderate group (MM, *n* = 23) with mild to moderate disease severity (BSA ≤ 50%) and a severe group (Sev., *n* = 60) (BSA > 50%). The MM group (75.48 ± 9.19 years) included 17 patients with cardiovascular disease and 2 with diabetes mellitus. In contrast, the Sev. group, averaging 71.78 ± 9.82 years, comprised 36 individuals with cardiovascular disease and 14 with diabetes mellitus (Table 2).

**TABLE 2:** Demographic, clinical, and serologic characteristics of the 2 disease severity subgroups.

Characteristics	MM n = 23	Sev. n = 60	*P*-value
Age, years, M ± SD	75.48 ± 9.19	71.78 ± 9.82	0.123a
**Sex, n (%)**			
Male	10 (43.48)	30 (50.00)	0.595b
Female	13 (56.52)	30 (50.00)	
**Medical history, n (%)**			
Cardiovascular disease	17 (73.91)	36 (60.00)	0.238b
Diabetes	2 (8.67)	14 (23.33)	0.229b
**Treatment, n (%)**			
Glucocorticoids/ immunomodulatory drugs	4 (17.40)	15 (25)	0.460b
Infection, n (%)	6 (26.10)	27 (45.00)	0.115b

a, Student’s *t*-test; b, Chi-square test; Treatment, medication use within 1 month prior to sampling; M, mean; SD, standard deviation; MM, patients with mild to moderate bullous pemphigoid; Sev., patients with severe bullous pemphigoid.

**FIGURE 2:**
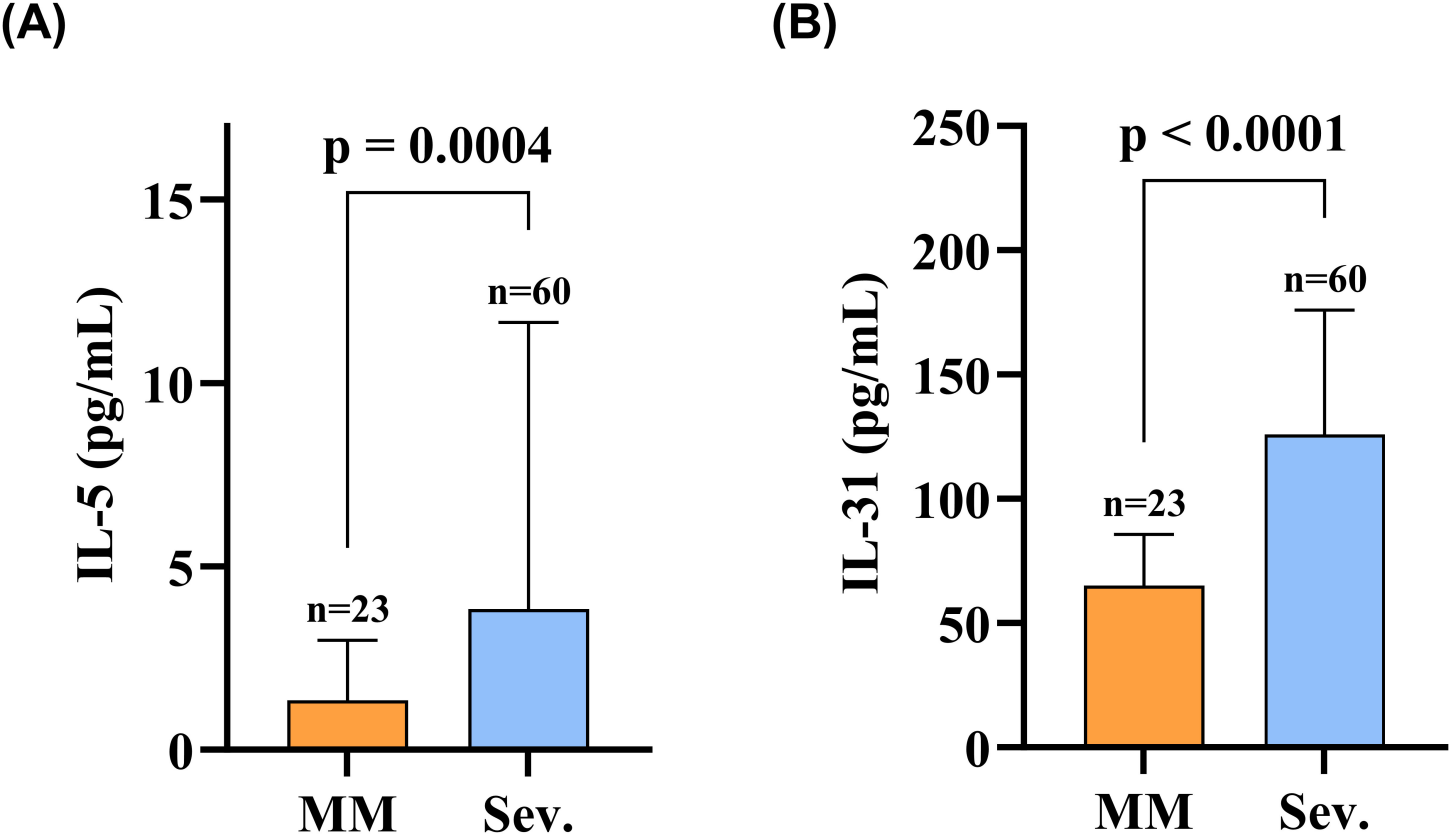
Serum IL-31 and IL-5 levels were elevated in patients in the Sev. group compared to the MM group. **(A)** Serum IL-5 levels in patients in the Sev. group compared with the MM group; **(B)** Serum IL-31 levels in patients in the Sev. group compared with the MM group. Statistical analysis was performed using the Mann-Whitney test, and the differences in serum IL-31 and IL-5 levels between the two groups were statistically significant (*p* < 0.05). MM, patients with mild-to-moderate BP; Sev., patients with severe BP; IL-31, interleukin 31; IL-5, interleukin 5.

Markedly higher serum levels of both IL-31 and IL-5 were observed in the Sev. group versus the MM group: IL-31 (Sev.: 125.99 [91.58–173.85] pg/mL vs. MM: 65.14 [51.27–85.47] pg/mL; *p* < 0.0001) and IL-5 (Sev.: 3.84 [1.57–10.63] pg/mL vs. MM: 1.35 [0.83–2.93] pg/mL; *p* < 0.001); this comparative profile is presented in Figure 2.

**FIGURE 3:**
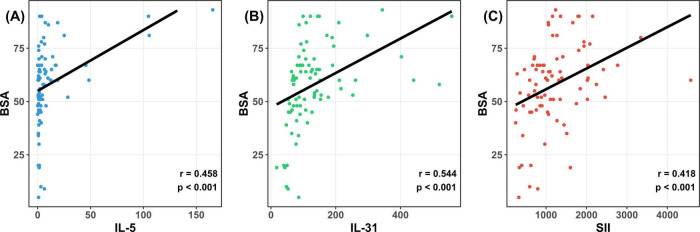
Systemic immunoinflammatory index (SII), IL-31, and IL-5 were positively correlated with BSA. **(A)** Correlation analysis between IL-5 and BSA in BP patients, some of the larger values of the scatter on the image are omitted; **(B)** correlation analysis between IL-31 and IL-31 in BP patients; **(C)** correlation analysis between SII and SII in BP patients. Using Spearman’s correlation analysis, IL-31 had the highest correlation coefficient, and the correlation between each index and BSA was statistically significant (*p* < 0.05). SII, systemic immunoinflammatory index; BSA, body surface area; IL-31, interleukin 31; IL-5, interleukin 5.

Subsequently, an analysis was conducted to ascertain the correlation between SII, IL-31, IL-5, and BSA. SII, which is calculated from peripheral blood neutrophil, platelet, and lymphocyte counts, is primarily utilized to evaluate the extent of systemic inflammation (21–23). The findings indicated a positive correlation between SII, IL-31, and IL-5 with BSA in patients with BP (Figure 3). The correlation coefficients were 0.418, 0.544, and 0.458, respectively, with the highest correlation coefficient observed between IL-31 and BSA. Detailed information on each index is shown in Table 3.

**TABLE 3:** Body surface area (BSA) correlation analysis.

Biomarkers	*R*-value	*P*-value	95% CI
SII	0.418	<0.001	0.216–0.594
IL-31	0.544	<0.001	0.359–0.681
IL-5	0.458	<0.001	0.285–0.604

Spearman correlation analysis was used; CI, confidence interval; SII, systemic immunoinflammatory index; IL-31, interleukin 31; IL-5, interleukin 5.

### 3.4 Logistic regression analysis of BP disease severity

We performed logistic regression analyses to assess the factors influencing disease exacerbation in BP. The findings from model 1 indicated that SII (*P* < 0.05, OR = 1.002, 95% CI: 1.001–1.003), IL-31 (*P* < 0.05, OR = 1.052, 95% CI: 1.025–1.080), and IL-5 (*P* < 0.05, OR = 1.335, 95% CI: 1.052–1.693) were identified as risk factors for BP disease exacerbation. Following the adjustment for confounders, including sex, age, cardiovascular disease, diabetes mellitus, medication status, and infection, both the SII and IL-31 metrics remained statistically significant in the model 2 and model 3. The results of Model 3 indicate that for every 100-unit increase in SII, the risk of disease progression increases by 49%; for every 1 pg/mL increase in IL-31, the risk of disease progression increases by 10%, thereby indicating that they function as independent factors that contribute to the exacerbation of BP disease (Table 4). Model validation showed: IL-31 and SII met linearity via Box-Tidwell test; Bootstrap internal validation revealed minimal overfitting (0.049). Calibration was excellent (Hosmer-Lemeshow *p* = 0.931; Brier Score = 0.078).

**TABLE 4:** Binary logistic regression model of BSA in patients with BP.

Biomarkers	Model 1		Model 2		Model 3		
	OR (95% CI)	*P*-value	OR (95% CI)	*P*-value	OR (95% CI)	*P*-value	VIF
IL-31	1.052 (1.025–1.080)	<0.001	1.087 (1.045–1.149)	<0.001	1.095 (1.037–1.157)	0.001	1.304
IL-5	1.335 (1.052–1.693)	0.017	1.443 (0.895–2.497)	0.132	1.439 (0.817–2.535)	0.207	1.274
SII	1.002 (1.001–1.003)	0.002	1.004 (1.002–1.007)	0.004	1.004 (1.001–1.007)	0.009	1.212

Model 1, unadjusted model; Model 2, adjusted for gender and age; Model 3, adjusted for cardiovascular disease, diabetes, immunomodulators, medication status, and infection.

### 3.5 Ability to predict BP disease activity by ROC curve analysis of biomarkers

**FIGURE 4:**
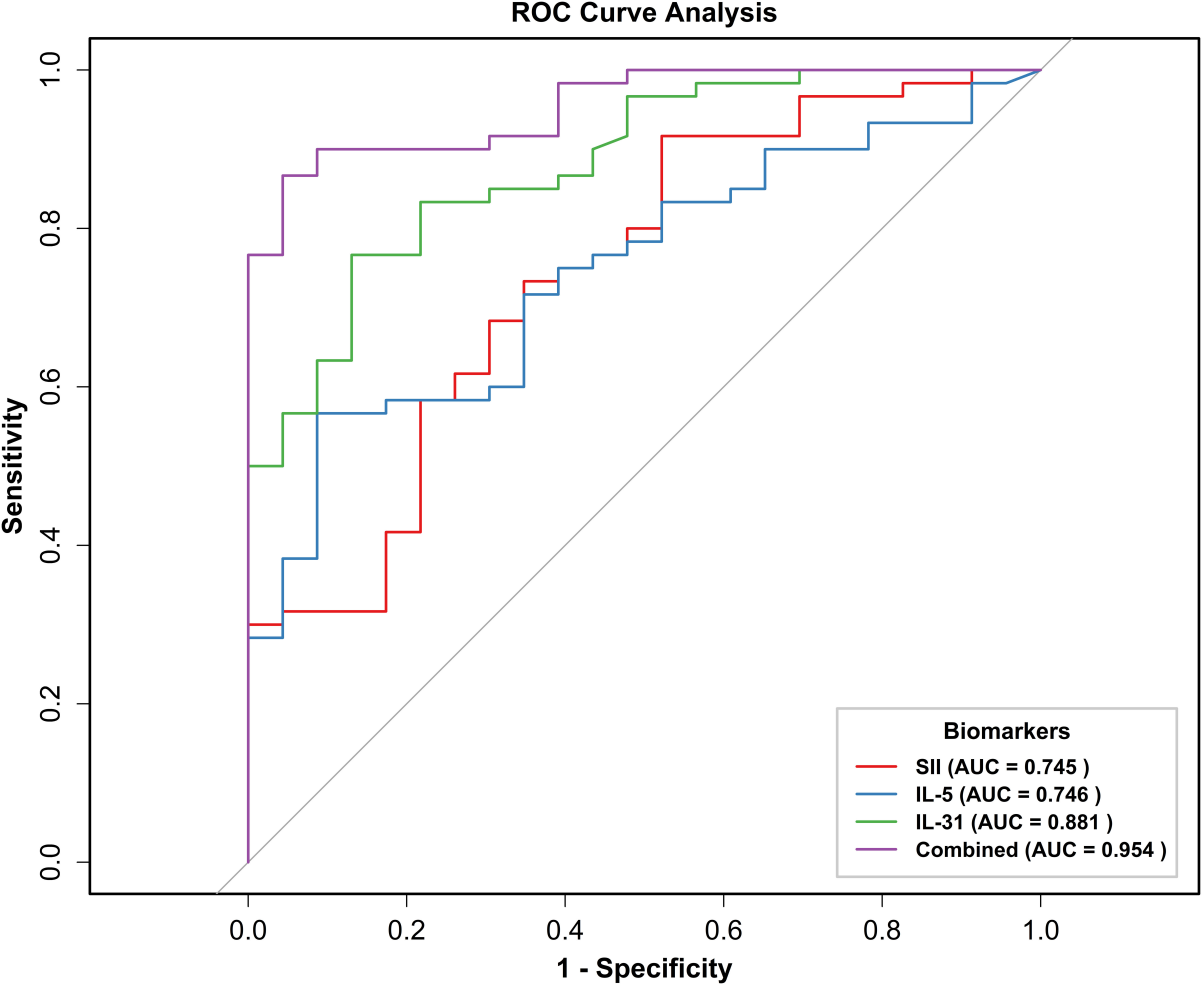
Receiver operating characteristic (ROC) curve of SII, IL-31, IL-5, and three indexes combined to predict BP exacerbation. ROC, subject operating characteristic curve; AUC, area under the curve; BSA, body surface area; SII, systemic immunoinflammatory index; IL-31, interleukin 31; IL-5, interleukin 5; Combined, combined prediction.

To assess the predictive ability of SII, IL-31, and IL-5 for BP severity, we plotted ROC curves (Figure 4). The results demonstrated that the area under the curve (AUC) predicted by SII, IL-31, IL-5, and the combination of these three metrics (Combined) were 0.745 (95% CI: 0.625–0.865), 0.881 (95% CI: 0.806–0.956), 0.746 (95% CI: 0.637–0.855), and 0.954 (95% CI: 0.914–0.993). The optimal critical values for SII, IL-31, and IL-5 were determined to be 657.04, 90.62, and 3.38, respectively. The sensitivity values were recorded as 0.92, 0.77, and 0.57, respectively, while the specificity values were documented as 0.48, 0.87, and 0.91, respectively.

### 3.6 Treatment of patients with BP resulted in a decrease in serum IL-31 levels

**FIGURE 5:**
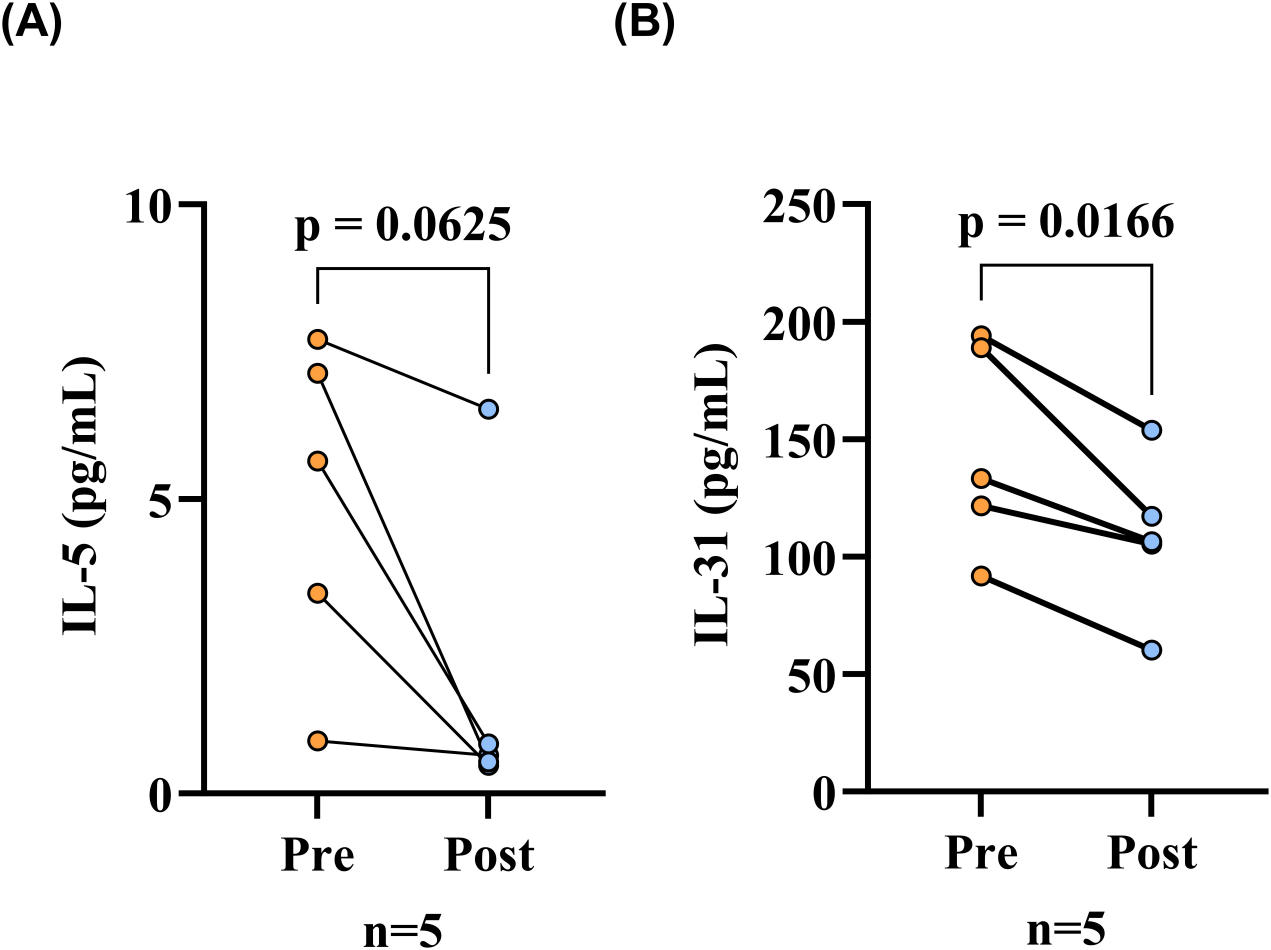
Serum IL-31 levels were reduced after treatment in BP patients. **(A)** Serum IL-5 levels before and after treatment in BP patients, comparison via the Wilcoxon signed-rank test; **(B)** Serum IL-31 levels before and after treatment in BP patients, comparison via paired *t*-tests. The differences in serum IL-31 levels before and after treatment of BP patients were statistically significant (*p* < 0.05), while the differences in serum IL-5 levels were not statistically significant. Pre, before patient treatment; Post, after patient treatment; IL-31, interleukin 31; IL-5, interleukin 5.

Finally, five BP patients were observed, and it was noted that all of the patients exhibited substantial control of their condition following treatment, as evidenced by the absence of new rash or healing of existing lesions (Table 5). The findings of the paired analysis demonstrated that the serum IL-31 level in the post-treatment (Post) group (108.62 ± 33.45 pg/mL) was significantly lower than the pre-treatment (Pre) baseline level (145.95 ± 44.25 pg/mL). This difference reached statistical significance (*p* = 0.017). Post-treatment (Post) serum IL-5 levels (0.64 [0.54–0.84] pg/mL) were lower than pre-treatment (Pre) baseline levels (5.64 [3.40–7.13] pg/mL), however, this decrease did not reach the threshold for statistical significance (*p* = 0.062, Figure 5).

**TABLE 5:** Demographic and clinical information of 5 follow-up patients.

Characteristics	Patients n = 5
Age, years, M ± SD	70.60 ± 5.22
**Sex, n (%)**	
Male	1 (20.00)
Female	4 (80.00)
**Treatment, n (%)**	
Glucocorticoids	5 (100.00)
Minocycline	2 (40.00)
Cyclosporine	1 (20.00)

## 4 Discussion

The primary goal of this research is to assess the relationship between serum levels of IL-31, IL-5, and SII and disease status among patients with bullous pemphigoid (BP). We compared IL-31 and IL-5 levels between BP patients and healthy individuals, between patients with different degrees of disease, and between patients before and after treatment. In addition, an investigation was conducted into the correlation of IL-31, IL-5, and SII with BP disease severity. The predictive ability of these indices for BP disease severity was also analyzed. Our findings indicated that serum levels of IL-31 and IL-5 were elevated in patients with BP when compared to healthy controls. Furthermore, we observed that serum levels of IL-31 and IL-5 were increased in the severe disease group compared to the mild-to-moderate group. Additionally, serum levels of IL-31 and IL-5 decreased in patients following treatment. Elevated levels of IL-31, IL-5, and SII were predictive of greater BP disease severity. The implications of our findings extend to the potential for enhanced prediction of the severity of BP disease and the development of new approaches to investigating BP treatment responses.

Interleukin-31 belongs to the gp130/IL-6 family of cytokines. It was originally described as a cytokine primarily produced by T cells. Research on numerous dermatologic conditions with pruritic symptoms, including BP, has demonstrated IL-31’s role as a significant driver of their pruritic responses. Chronic urticaria and psoriasis are associated with elevated serum IL-31 levels (10, 24–27). In the present study, elevated serum levels of IL-31 (103.61 [77.42, 146.77] pg/mL) were observed in patients as compared to healthy controls (86.02 [51.49, 122.53] pg/mL). This finding aligns with prior evidence (7, 28).

Prior studies on the association between IL-31 and BP have predominantly centered on the relationship between IL-31 and pruritus; however, there is increasing evidence of the pro-inflammatory effects of IL-31, which has been found to be associated with the activity of some inflammatory skin diseases (14–16). IL-31 has been reported to promote helper Th2 cell-driven inflammation and stimulate eosinophil activation, suggesting a potential link between IL-31 and bullous pemphigoid, which has Th2-driven inflammation and eosinophil infiltration as a pathologic mechanism (11–13). The body surface area of lesions (BSA) was assessed in patients with BP as a proxy for disease severity. Patients with BP were then classified into mild-moderate and severe groups based on their BSA (20). Our findings indicated that patients with BP in the severe category exhibited significantly elevated serum IL-31 levels (125.99 [91.58, 173.85] pg/ml) in comparison to those observed in the mild-moderate group (65.14 [51.27, 85.47] pg/ml). Moreover, serum IL-31 levels correlated positively with BSA (*r* = 0.544). IL-31 is primarily a Th2 cell-derived cytokine. It can act on the IL-31R on eosinophils, causing eosinophil activation (13). The influence of several eosinophil functions on the pathogenesis of BP has been shown, and studies have demonstrated that degranulation of eosinophils is critical to the formation of BP blisters; Furthermore, the secretion of toxic granule proteins by eosinophils results in local inflammatory responses that intensify the patient’s symptoms, and therefore, IL-31 exacerbates the severity of BP through the activation of eosinophils. This finding provides a potential explanation for the observed relationship between IL-31 and the disease activity of BP (29, 30). Subsequently, a logistic regression analysis of IL-31 and disease severity was conducted, which indicated that IL-31 was a risk factor for BP disease exacerbation. Following adjustment for confounders, IL-31 (OR = 1.095, 95% CI: 1.037–1.157) remained significant (*P* < 0.05), suggesting that IL-31 was an independent influencing factor. Finally, we examined IL-31 and IL-5 levels pre- and post-treatment in five patients. This evaluation demonstrated a significant decrease in serum IL-31 after treatment, suggesting the potential use of IL-31 in assessing the response to BP treatment. Serum IL-5 levels exhibited a downward trend in the patient cohort post-treatment, though this observation lacked statistical significance. This outcome may be constrained by the modest sample size (*n* = 5).

Interleukin-5, a cytokine secreted primarily by Th2 cells, exhibited significantly elevated levels in serum samples from BP patients (2.86 [1.22, 7.10] pg/mL) compared to healthy control subjects (0.79 [0.65, 1.00] pg/mL). This observation aligns with previously reported findings in the literature, which documented an abnormally elevated profile of IL-5 in serum samples (31, 32). Indeed, interleukin-5 (IL-5) has been demonstrated to correlate with eosinophil chemotaxis and activation, and it has been shown to induce dermal-epidermal junction detachment in patients with BP through the production of matrix metalloproteinase 9 (MMP9). Furthermore, previous studies have observed a positive association between IL-5 levels in blister fluid of BP patients and the number of lesions present in these patients (3, 5, 9). In the present study, a positive correlation was observed between the serum IL-5 levels of BP patients and the area of lesions of the patients (*r* = 0.458). In the unadjusted model of logistic regression analysis, IL-5 demonstrated a significant predictive value for disease severity (OR = 1.335, 95% CI: 1.052–1.693), thereby suggesting its biological significance as a core modulator of Th2-type immune responses.

However, the independent predictive efficacy of IL-5 was lost after the introduction of IL-31 and systemic immunoinflammatory index (SII) in the crude and refined models (*p* > 0.05), and IL-5 and IL-31 were found to be correlated in our study (*r* = 0.352, *p* < 0.05), which may reflect the role of both in the Th2-type immune pathway by a synergistic mechanism, and we hypothesized that this overlapping of biological functions may have led to a partial dilution of the effect of IL-5 by IL-31 in the multifactorial model, weakening its statistical significance. Despite the loss of significance of IL-5 in the multifactorial model, its clinical value as a marker of Th2-type immune response and eosinophil activation remains, and anti-IL-5 monoclonal antibody may ameliorate cutaneous lesions by decreasing eosinophil levels, whereas co-targeting of IL-31 may optimize efficacy (33).

Systemic immunoinflammatory index is an index utilized to evaluate the extent of systemic inflammation in an organism. This index is derived from the peripheral blood platelet count, neutrophil count, and lymphocyte count. A mounting body of research has demonstrated the potential of this index to serve as a prognostic tool, capable of anticipating the severity of specific diseases and the effectiveness of therapeutic interventions (17–19). The SII has been linked to the severity of several autoimmune diseases. Reports have suggested that the SII may serve as a prognostic tool to evaluate the severity and disease activity of several immune-mediated conditions, including psoriasis, rheumatoid arthritis (RA), and systemic lupus erythematosus (SLE) (21, 23, 34, 35). Therefore, we included this index in our study. The findings of the present study indicated that patients with elevated SII values exhibited a greater extent of lesions (*r* = 0.418). In the logistic regression models, following adjustments for age, gender, cardiovascular disease, and diabetes, SII (OR = 1.004, 95% CI: 1.001–1.008) retained statistical significance (*p* < 0.05). This finding indicates that SII functions as an independent predictor of disease exacerbation in patients with BP.

Finally, ROC curves were developed to assess the predictive capacity of IL-31, IL-5, and SII for disease severity. The findings of this study indicated that interleukin-31 (IL-31), IL-5, and SII exhibited a high degree of reliability in predicting disease severity. Specifically, IL-31 (AUC: 0.881, 95% CI: 0.806–0.956) demonstrated a superior predictive capacity for disease severity when compared with SII (AUC: 0.745) and IL-5 (AUC: 0.746). Furthermore, the combination of these three metrics resulted in an enhanced predictive value.

The present study has certain limitations. First, due to study limitations, we used skin lesion area as the basis for severity stratification, which, although clinically actionable, did not incorporate a combination of mucosal involvement and itchiness in the BPDAI score, potentially overlooking the impact of other features of the disease on inflammatory markers. Secondly, given the relatively small number of patients included in the study, further validation of the findings in larger, multicenter, high-quality clinical studies is necessary in the future. Secondly, given the relatively small number of patients included in the study, further validation of the findings in larger, multicenter, high-quality clinical studies is necessary in the future.

## 5 Conclusion

To conclude, a systematic investigation was conducted to explore the correlation of IL-31, IL-5, and SII with BP onset, disease severity, and response to treatment. The findings of this study indicated a positive correlation between IL-5, IL-31, and SII and the severity of the disease in patients with BP. Furthermore, the study demonstrated that these markers exhibited a satisfactory predictive capacity for the exacerbation of BP disease. Consequently, IL-31, IL-5, and SII can serve as predictive indicators of BP disease severity, facilitating disease progression assessment and offering novel opportunities for biomarkers to evaluate BP treatment response.

## Data Availability

The raw data supporting the conclusions of this article will be made available by the authors, without undue reservation.
